# Activated allograft combined with induced membrane technique for the reconstruction of infected segmental bone defects

**DOI:** 10.1038/s41598-024-63202-9

**Published:** 2024-06-01

**Authors:** Xiaohua Wang, Chao Jia, Hongri Wu, Fei Luo, Tianyong Hou, Gang Li, Sien Lin, Zhao Xie

**Affiliations:** 1 Department of Orthopaedics, First Affiliated Hospital, Third Military Medical University (Army Medical University), Chongqing, 400038 People’s Republic of China; 2 Department of Orthopaedics, Navy 905 Hospital, Navy Medical University, Shanghai, People’s Republic of China; 3 Stem Cells and Regenerative Medicine Laboratory, Li Ka Shing Institute of Health Sciences, The Chinese University of Hong Kong, Prince of Wales Hospital, Shatin, Hong Kong SAR People’s Republic of China; 4 Musculoskeletal Research Laboratory, Department of Orthopaedics & Traumatology, Faculty of Medicine, The Chinese University of Hong Kong, Prince of Wales Hospital, Shatin, Hong Kong SAR People’s Republic of China

**Keywords:** Trauma, Outcomes research

## Abstract

This study was desinged to evaluate the efficacy and safety of activated allograft combined with the induced membrane technique for reconstruction of infected segment bone defects of lower limbs. A retrospective analysis was conducted on 19 patients from May 2015 to February 2017. After debridements, the bone defects were filled with antibiotic bone cement to form the induced membrane. Autologous mesenchymal stem cells were seeded onto allografts to construct activated allograft, which was implanted into the induced membrane after infection was controlled. The clinical efficacy and complications were observed. 19 patients with 20 infected segment bone defect were evaluated. The average deficit size was 11.08 (4–17) cm in length. After a mean follow-up of 71.84 (61–82) months, bone union was achieved in 16 patients (17 sites), resulting in a final union rate of 84.21% (16/19 patients). The average bone union time was 10.18 (5–28) months. There were 2 patients with recurrence of infection, 3 patients with graft absorption, and 1 patient with malunion due to implant breakage. There were no graft-related complications. This study provides clinical significance for the treatment of patients with insufficient autologous bone.

## Introduction

Although significant progress has been made in bone defect treatments, the clinical treatment efficacy of large bone defects remains an area that requires further optimized. Induced membrane technique is a widely used strategy for bone defects due to its simple operation process and few complications^1–3^. Although induced membrane provide a variety of osteogenic and vascular factors for autologous cancellous bone, which can promote the rapid healing of bone defects^4,5^. One of the difficulties of the induced membrane technique is that the total amount of autologous cancellous bone in patients cannot meet the needs of large segmental bone defects^6^. The use of allogeneic for the treatment of bone defects is not very effective in patients with insufficient autologous bone mass or unwillingness to accept complications in the autologous bone donor area. Activated allograft, which simulates the structure and function of autologous bone through seeding autologous osteogenesis precursor cell on a scaffold, is expected to solve this problem. Although significant progress has been made in basic research on activated allograft, it has not been widely used in clinical practice. In this study, we report a series of patients with infected segment bone defects of lower limbs treated with activated allograft combined with the induced membrane technique. Our aim was to investigate the outcomes of this new method and to provide a reference for clinicians when applying this method.

## Patients and methods

We restrospectively analysed medical records of patients with large segmental bone defects caused by bone infection in our deparment between May 2015 and February 2017. The Ethics Committee of the First Affiliated Hospital of the Army Medical University approved this retrospective investigation, and all methods were performed in accordance with the relevant guidelines and regulations. The need of informed consent was waived by The Ethics Committee of the First Affiliated Hospital of the Army Medical University due to retrospective nature of the study. The inclusion criteria were: (1) Lower extremity bone infection patients admitted to our department; (2) Segment bone defects > 3 cm; (3) Patients with insufficient autologous cancellous bone; (4) Treatment with activated allograft combined with induced membrane technique; (5) Age < 60 years; (6) Follow-up time > 5 years. The exclusion criteria were: (1) Infections without curative treatment (palliative care); (2) Patients with malignant diseases; (3) Serious vascular and nerve injuries with no possibility of limb salvage.

## Surgical technique

**Figure 1:**
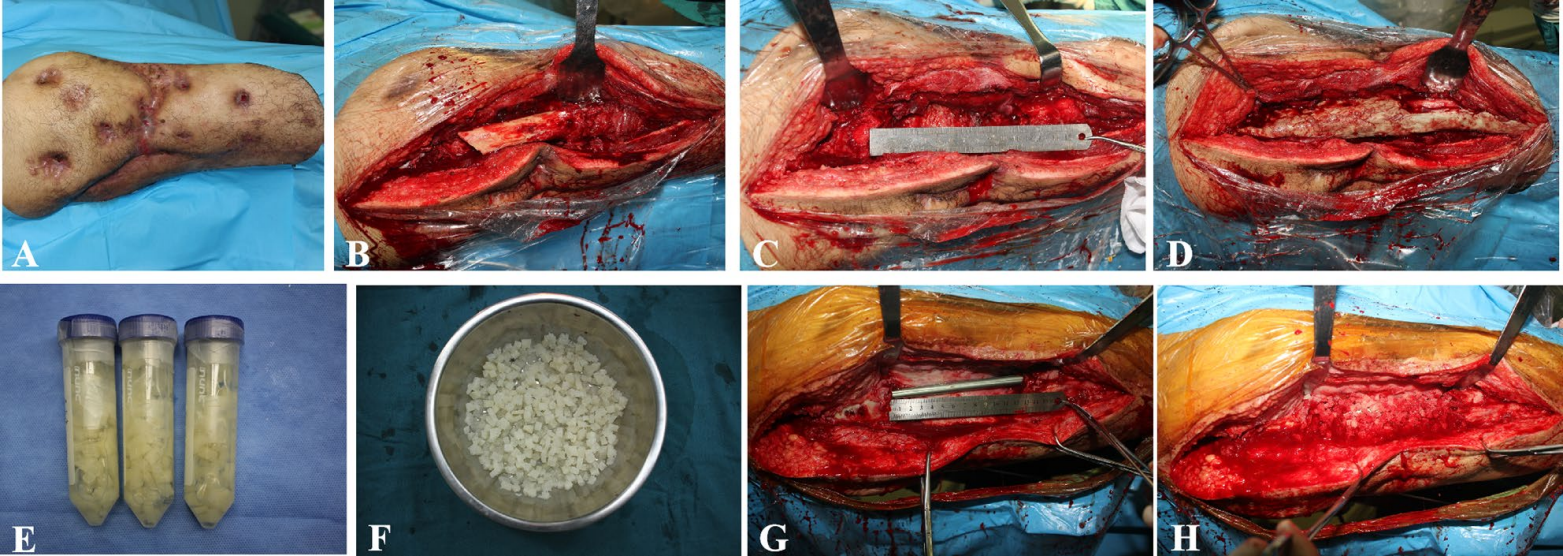
(**A**) Preoperative photo; (**B**) Infected soft tissue and bone tissue; (**C**) After debridement; (**D**) Antibiotic bone cement filled with the defects and wrapped the fixation (locking plate); (**E**,**F**) Activated allograft; (**G**) Intramedullary nails are used to fix the bone ends in the bone reconstruction stage; (**H**) Implantation of Activated allograft.

In the first stage, the bone defects were filled with antibiotic bone cement in order to eradicate infection and form induced membrane after thorough debridement. In the second stage, activated allograft bone was implanted in the induced membrane after the removal of antibiotic bone cement to reconstruct bone defects (Fig. 1).

### Debridement

The infection site and range of debridement were determined by imaging results of magnetic resonance and bone scanning preoperatively. The sinus, infected bone and surrounding necrotic tissue were debrided and samples were taken for bacterial culture and pathological examination. After debridement, antibiotic bone cement (5 g vancomycin per 40 g bone cement) were implanted to fill the remained bone defects (Fig. 1D). Sensitive antibiotics were given for 2 weeks according to bacterial culture results and drug sensitivity results.

### Preparation of activated allograft bone

**Figure 2:**
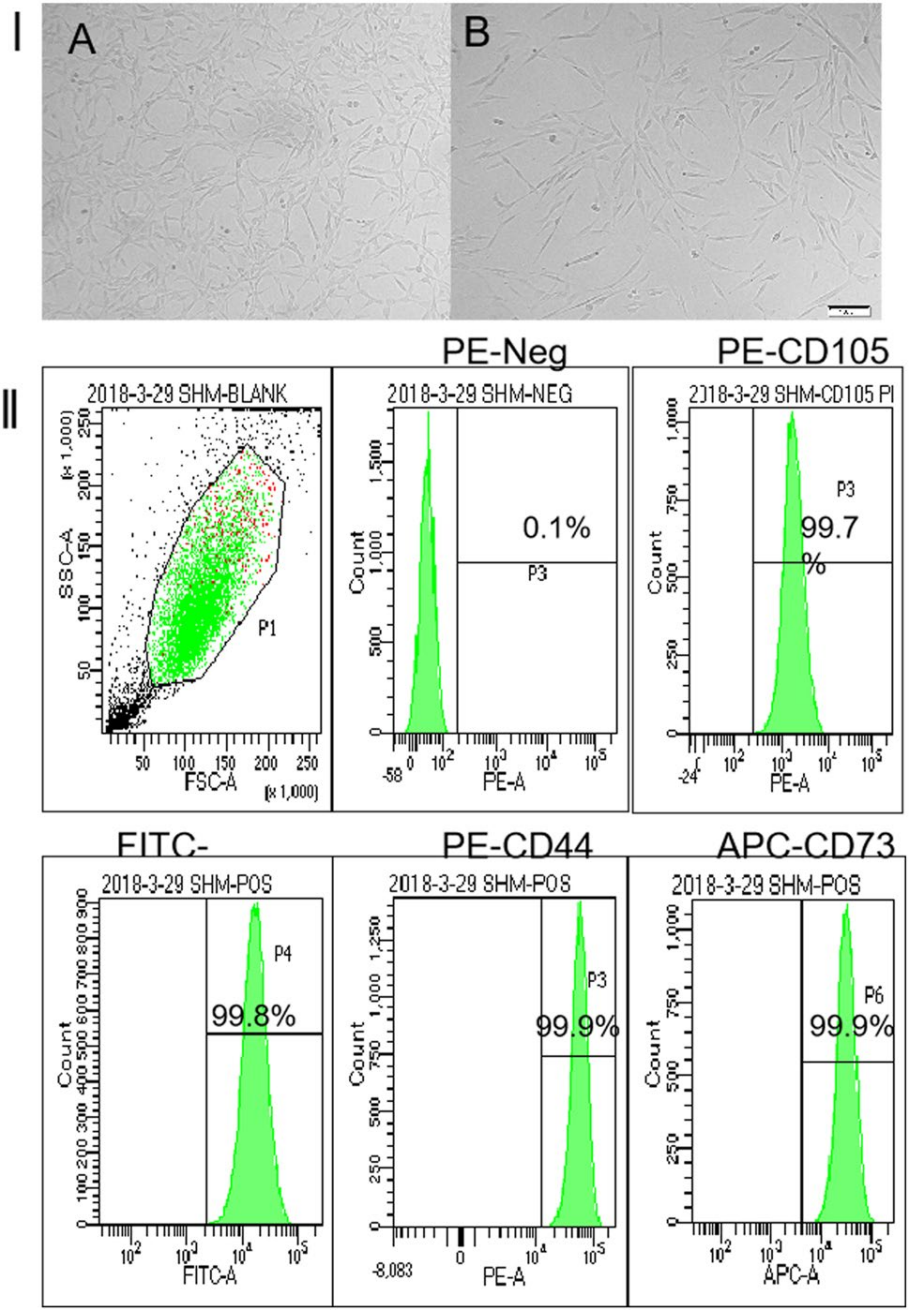
Panel I. Morphology of BMSCs cultured in Vitro. (**A**) Cells at passage 1; (**B**) Cells at passage 3; Panel II. Flow cytometric analysis of markers characteristic for BMSCs. Expression of the markers CD44, CD73, CD90 and CD105 was more than 99%; The negative percentages of BMSCs were 0.1%.

**Figure 3:**
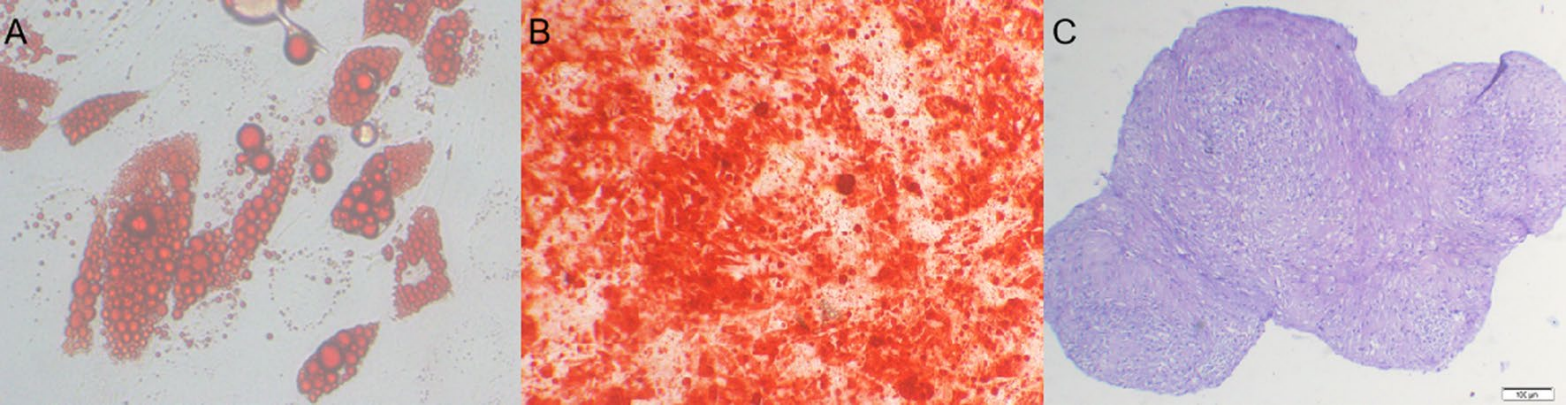
Detected the differentiation capacity of the BMSCs. (**A**) Adipogenic (Oil Red staining); (**B**) osteogenic (Alizarin Red staining); (**C**): Chondrogenic differentiation potential (Alcian blue staining).

**Figure 4:**
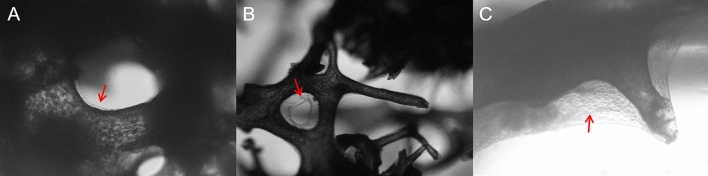
In vitro culture of bone scaffolds inoculated with BMSCs (red arrows).

Assessment of systemic or local infection eradication: (1) no swelling, pain, redness and pus at the infection site; (2) serum inflammation index declined to normal, (3) no active infection in the whole body; (4) the imaging examination did not indicate infection. A total of 50 ml of autologous bone marrow was taken from multi-site of the anterior or posterior iliac crests and bone marrow derived MSCs (BMSCs) were isolated. The surface markers of BMSCs were determined by flow cytometry (Fig. 2). Antibodies including FITC-labeled CD90, APC-labeled CD73, PE-labeled CD44, percp-cy5.5-labeled CD105 and PE-labeled CD19 + /CD11b + /CD34 + /CDHLa-DR + mixed antibodies (all purchased from BD) were used. The differentiation capacity of the BMSCs were further determined by specific staining (Fig. 3). Allogeneic bone (Beijing Daxing Biotechnology Co., Ltd) was cut to a diameter of 0.3 cm × 0.3 cm × 0.3 cm, then immersed in the BMSCs culture medium. 1 ml BMSCs suspension in a density of 1 × 10^7^/ml were inoculated into 4 cm^3^ of bone tissue, then cultured for 3 days. Morphology of the stem cells in scaffolds were observed under a microscope (Fig. 4). To ensure their safety, the scaffold underwent testing for the presence of bacteria, endotoxin, and mycoplasma, and was only implanted following negative results. The bone graft material was washed with sterilized saline and implanted into the bone defects.

### Bone grafting

Before bone grafting, the length and diameter of the bone defects were measured by CT scan, and then estimate the volume of the bone defects (V = πR^2^ × H according to cylinder, π = 3.14, R is the radius of the bone defects, H is the length of the bone defect), plus 30% of the calculated volume for the actual implantation. The bone cements were removed after 8 weeks. The surrounding induced membrane will be protected during the secondary operation. After performing a rapid pathological examination intraoperatively in frozen sections, only patients who were determined to not have suppurative inflammation were eligible for grafting. Sensitive antibiotics were used prophylactively in the second stage according to the results of intraoperative bacterial culture. During the bone reconstruction stage, intramedullary nails are used to fix the bone ends (Fig. 1G). The defects were fully filled with activated allograft bone. The induced membrane was sutured carefully, drainage was left and incision was closed after grafting.

### Postoperative follow-up

Follow-up was performed every month after the first stage and every 3 to 6 months after the second stage. The review included clinical symptoms and signs of infection (such as swelling, fever, pain and sinus formation), laboratory examinations (WBC, ESR, CRP). The recurrence of infection, bone healing and complications were observed and recorded. Infection control was defined as the absence of both clinical symptoms and normalisation of laboratory inflammatory markers throughout the visits. The bone union time was obtained by X ray. Bone union was defined as three-sided cortical bridging on two perpendicular X-rays of the defect zone^7^. After X-ray examination reveals the formation of callus, weight-bearing exercises can be initiated, and the weight can be gradually increased.

## Results

19 patients with 20 infected segment bone defects were enrolled in this study, including 14 males and 5 females with an average age of 28 (5–52) years. According to the aetiological calssification proposed by Waldvogel, there were 4 cases of haematogenous osteomylelitis and 15 cases of post-traumatic osteomyelitis. The duration of bone infection before admission ranged from 1 to 250 months, and an average of 4.79 surgical procedures were performed.

After thorough debridement, there were residual segmental bone defects in 13 femurs and 7 tibias, with one patient involving both the femur and tibia. The average length of bone defects was 11.08 (4–17) cm. Among the 19 patients, 15 (78.95%) had positive bacterial isolations. Among these cases, 7 (46.7%) cases infected with Staphylococcus aureus (including 5 with methicillin-resistant *Staphylococcus aureus*), 5 with *Escherichia coli*, 3 with *Pseudomonas aeruginosa*, 1 with *Enterococcus faecalis*, and 3 other types. In 4 remaining cases, no bacteria were isolated (data shown in Table 1). The average bone graft volume was 154 (50–350) cm^3^.

**Table 1:** Patient demographics.

Patient number/sex/age (y)	Aetiological calssification of osteomyelitis	Location	Duration of infection (M)	Bacterium	BD length (cm)	Bone grafting (ml)	Infection control (Y/N)	Bone union time (M)	Complication
1/M/5	Post-traumatic	Femur	1	Escherichia coli	10	50	Y	5	None
2/M/30	Post-traumatic	Femur	3	*Escherichia coli*, *Enterococcus faecalis*	10	120	Y	10	Graft absorption
3/M/52	Post-traumatic	Tibia	4	*Staphylococcus arlettae*	12	200	Y	11	Implant breakage, Malunion
4/F/39	Post-traumatic	Tibia	6	*Stenotrophomonas aerophila*	4	70	Y	28	None
5/M/17	Post-traumatic	Femur	8	None	10.5	180	Y	7	None
6/M/13	Post-traumatic	Femur/Tibia	2	Methicillin-susceptible* Staphylococcus aureus*, *Escherichia coli*	6/14	100/100	Y	5/7	Foot deformity
7/M/40	Post-traumatic	Femur	9	*Escherichia coli*, *Pseudomonas aeruginosa*	11	150	Y	15	None
8/M/26	Post-traumatic	Tibia	12	*Staphylococcus epidermidis*	16	85	Y	18	None
9/M/10	Haematogenous	Femur	3	Methicillin-resistant* Staphylococcus aureus*	17	200	Y	8	None
10/F/44	Post-traumatic	Femur	5	Methicillin-susceptible* Staphylococcus aureus*, *Pseudomonas aeruginosa*	7	150	Y	7	None
11/F/45	Post-traumatic	Tibia	22	*Escherichia coli*	16	210	N	Amputation	None, Infection recurrent
12/M/20	Post-traumatic	Femur	28	*Methicillin-resistant staphylococcus aureus*	5	70	Y	8	None
13/M/26	Post-traumatic	Femur	65	Methicillin-resistant* Staphylococcus aureus*	17	350	Y	10	None
14/F/20	Haematogenous	Femur	121	Methicillin-resistant* Staphylococcus aureus*	15	200	Y	12	Graft absorption
15/F/33	Haematogenous	Femur	250	Methicillin-resistant* Staphylococcus aureus*	15	120	Y	7	Graft absorption
16/M/17	Post-traumatic	Tibia	24	*Pseudomonas aeruginosa*	6	150	Y	9	None
17/M/27	Haematogenous	Femur	12	None	8.5	150	Y	-	Nonunion
18/M/37	Post-traumatic	Femur	9	None	15.5	275	Y	6	None
19/M/31	Post-traumatic	Tibia	9	None	13	150	N	–	Infection recurrent

**Figure 5:**
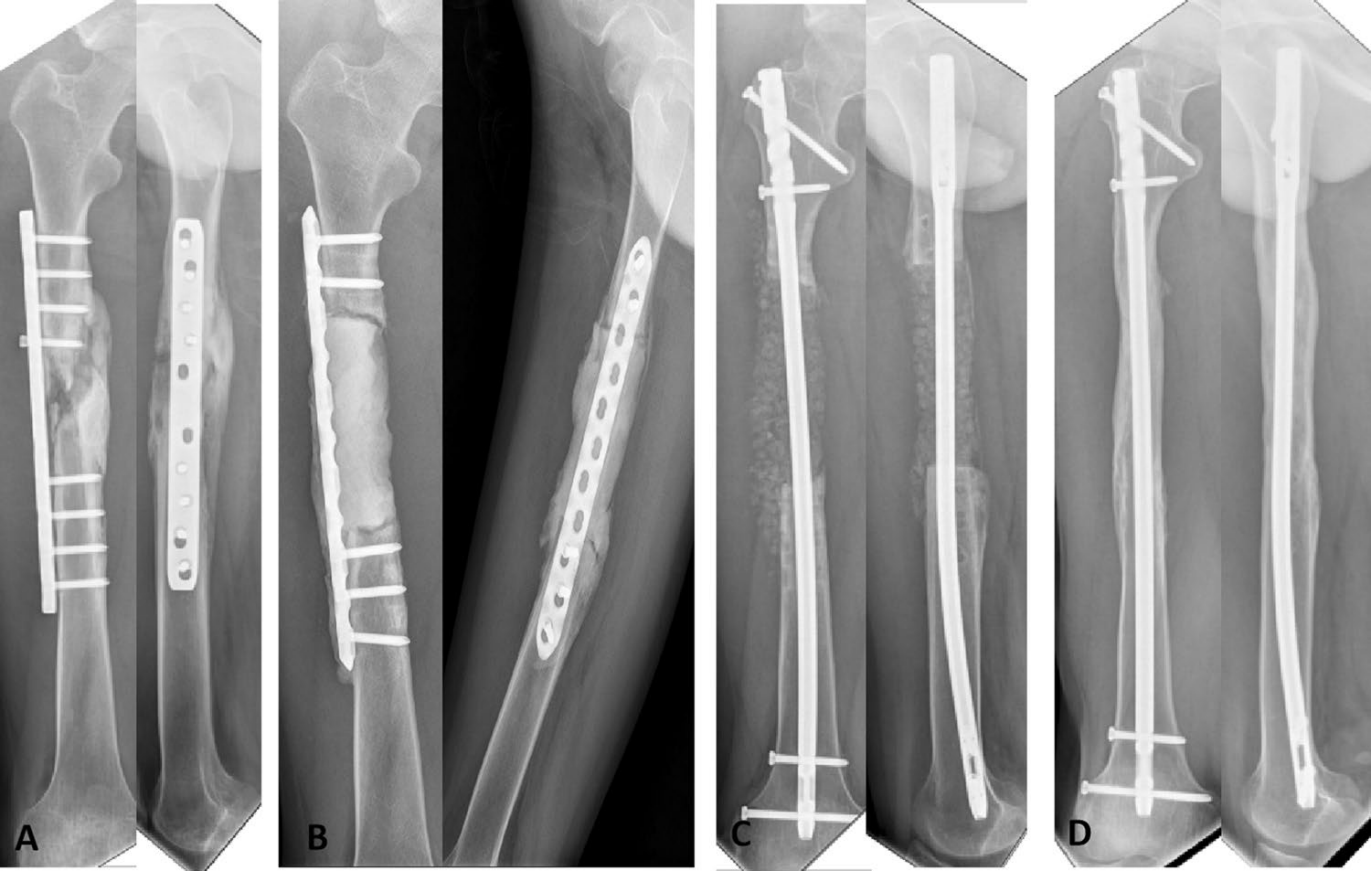
Typical case: Male, 17Y, repeated draining fistula of the right femur for 8 months, bone defect 10.5 cm. (**A**) Preoperative X rays; (**B**) 10.5 cm bone defects were implanted with PMMA cements; (**C**) X-rays of the bone defects 1 month after grafting; (**D**) X-rays showed bone union after 18 months.

After a mean follow-up of 71.84 (61–82) months, bone union was achieved in 16 patients (17 sites), resulting a final union rate of 84.21% (16/19 patients). The average time to bone union was 10.18 (5–28) months. There was recurrence of infection in 2 (12.5%) patients. One patients refused further treatment and opted for amputation, the other was treated with bone transport technique. Additionally, three cases with graft absorption required further autografting. Malunion due to implant breakage occurred in one case. During the follow-up period, there were no graft-related complications. (Typical case is presented in Fig. 5).

## Discussions

Treatment of infected bone defects is challenging, and the ultimate goal is to clear the infection and restore limb function. Cierny-Mader type IV osteomyelitis requires thorough debridement and often results in bone defects when the infection is controlled. To solve this problem, various techniques have been developed, such as the Illizarov technique. Since its report in 2000, induced membrane technology has gradually gained popularity and been gradually promoted in clinical applications in recent years^8^. Induced membrane technique has been used to repair infected bone defects in extremities in our department since 2012, and the early clinical effect is good^9,10^. However, the lack of autologous bone graft is still one of the difficulties in repairing large segmental bone defects with membrane induction technique.

Autogenous cancellous bone graft has long been considered the gold standard for repairing bone defects due to its fast repair and low revision rate compared with other materials. However, the limited availability of autogenous bone and potential donor site complications limit its use in clinical treatment^11,12^. Therefore, researchers began to search for materials that could replace autologous cancellous bone.

Tissue-engineered grafts have shown promise as an alternative to autografts^13^. In 2001, Quarto reported successful treatment of 3 cases of bone defects using tissue-engineered grafts constructed with MSCs and macroporous hydroxyapatite scaffolds^14^. In 2010, Eric successfully repaired a 7.2 cm bone defect using a novel multiple disc graft constructed with autologous mesenchymal stem cells and calf bone in a 58-year-old patient^15^. Although these cases pioneered the use of tissue engineered grafts to treat bone defects, they are more commonly used at nonbearing sites, such as facial and cranial bones, and the extent of bone defects is modest^16,17^. In 2010, Maik reported that allogenic cancellous bone seeded with human mesenchymal stromal cells has the potential to repair bone defects, but it has not been widely used in clinical practice^18^. In this study, the induced membrane technique combined with activated allogeneic bone was applied to the clinical treatment of infectious bone defects. We followed up with patients for an average of more than 5 years and found that a final union rate of 84.21% (16/19 patients).

Osteogenesis, osteoinduction and osteoconduction of the graft are particularly important for bone defects repair^13,19^. Although autograft has long been considered the gold standard, they are limited to small bone defects^20^. For large segmental bone defects, autologous bone is prone to bone resorption, suggesting special requirements for osteogenic environment. Induced membrane technology provides an osteogenic microenvironment rich in stem cells and vascular cells for the graft, which is an effective method to repair bone defects^1,5^. Activated allogeneic bone is one of the popular alternative materials in recent years, but its clinical application is limited due to the difficulty of vascularized construction in vivo. The process of bone healing is controlled by engineering adult stem cells to express genes like bone morphogenetic proteins, core binding factor α1 (Cbfa1), vascular endothelial growth factor (VEGF)^21^. Induced membranes are constructed in vivo using perfusion bioreactors and serve as a mechanical barrier to prevent reabsorption of the contained graft. Most importantly, the induced membrane provided a large amount of osteoinductive factors, growth factors, stem cells, and vascular cells that can promote revascularization and osteogenesis^22^. In this study, we used the induced membrane technique combined with activated allograft bone seeded with autologous stem cells to repair large segmental infected bone defects. The bone union rate is 84.21% (16/19 patients) and the overall efficacy was reliable. It suggests that the induced membrane plays an important role in the early stage of bone defects reconstrution, possibly due to vascularization of the graft. However, the average healing time was 10.18 (5–28) months in this group, which was longer than that in autogenous bone group^10^. These results suggest the importance of graft materials in osseointegration and osteogenesis in repairing large segmental bone defects.

The goal of a successful bone graft is to restore the anatomical, physiological and functional status of the bone^23^. Activated allograft bone involves the use of osteoconductive biomaterial scaffolds with osteogenic cell populations and osteoinductive bioactive factors^24^. The scaffolds should have appropriate shape, size, and mechanical competence^25^ and serve as a template for cell interactions and the formation of bone extra cellular matrix to provide structural support to the newly formed tissue^24,26^. While various synthetic biomaterials like inorganic ceramics metals, and synthetic biodegradable polymer composites have been investigated for their potential as bone scaffold materials^24^, their common drawback is inherent brittleness^27^. In this study, we selected allogeneic bone as scaffold material, which is structurally closest to autogenous bone and has high osteoinductive potential and remodeling characteristics^28,29^, making it an ideal tissue-engineered scaffold material. Bone marrow is a multipotent stem cell reservoir of mesenchymal tissue that can differentiate into fibroblasts, osteoblasts, adipocytes and reticular cells^30^. In the present study, we isolated autologous BMSCs and seeded them in scaffolds to construct individual tissue-engineered bone, which has certain advantages, such as accessibility, lack of ethical controversies, painless for donors.

In the present study, we observed graft absorption in three patients. To prevent this, we recommend that the bone graft volume should be at least 120% greater than or equeal to the bone defect volume. Although activated allograft has shown promise for reconstructing large segmental bone defects, it showed longer union time compared to autografts. Therefore, in future research, we should focus on how to improve the activity of seeded cells to accelerate bone uniou, as well as determine the appropriate age, site, and clinical application indications.

Our study highlights the importance of graft materials in the reconstruction of large segmental bone defects and the potential of activated allografts as alternatives to autografts. However, there are still some limitations in this study. First, this is a preliminary clinical study, and the mechanisms behind the observed effect needs further in-depth investigation and discussion. For example, there is a lack of data on the biomechanical stability of the created bone materials. Second, our activated allogeneic bone simply seeded stem cells on the scaffold without adding growth factors to improve its clinical efficacy, which may contribute to the prolonged bone healing time. Third, the source of allogeneic bone is limited and the technical requirements are high. This limits its widespread clinical application and can only be applied in some qualified hospitals. Fourth, there is a lack of comparison with other methods.

## Conclusion

Activated allograft combined with the induced membrane technique is a safe and reliable method for the treatment of large infected segmental bone defects in the lower extremity.

## Data Availability

The datasets used and/or analysed during the current study available from the corresponding author on reasonable request.
